# Effects Mediated by Dimethyl Fumarate on In Vitro Oligodendrocytes: Implications in Multiple Sclerosis

**DOI:** 10.3390/ijms23073615

**Published:** 2022-03-25

**Authors:** Claudia Guerriero, Giulia Puliatti, Tamara Di Marino, Ada Maria Tata

**Affiliations:** 1 Department of Biology and Biotechnologies Charles Darwin, Sapienza University of Rome, 00185 Rome, Italy; claudia.guerriero@uniroma1.it (C.G.); giu.puliatti@gmail.com (G.P.); tamaradimarino@outlook.it (T.D.M.); 2 Research Centre of Neurobiology Daniel Bovet, 00185 Rome, Italy

**Keywords:** dimethyl fumarate, oligodendrocytes, Oli neu cell line, oxidative stress, lipids, multiple sclerosis

## Abstract

Background: Dimethyl fumarate (DMF) is a drug currently in use in oral therapy for the treatment of relapsing-remitting multiple sclerosis (RRMS) due to its immunomodulatory and neuroprotective effects. The mechanisms by which DMF exerts its therapeutic effects in MS and in particular its influence on the oligodendrocytes (OLs) survival or differentiation have not yet been fully understood. Methods: Characterization of Oli neu cells was performed by immunocytochemistry and RT-PCR. The effect of DMF on cell proliferation and morphology was assessed by MTT assay, trypan blue staining, RT-PCR and Western blot analysis. The antioxidant and anti-inflammatory properties of DMF were analysed by ROS detection through DCFDA staining and lipid content analysis by Oil Red O staining and TLC. Results: DMF has been observed to induce a slowdown of cell proliferation, favoring the oligodendrocyte lineage cells (OLCs) differentiation. DMF has an antioxidant effect and is able to modify the lipid content even after the LPS-mediated inflammatory stimulus in Oli neu cells. Conclusions: The results obtained confirm that DMF has anti-inflammatory and antioxidant effects also on Oli neu cells. Interestingly, it appears to promote the OLCs differentiation towards mature and potentially myelinating cells.

## 1. Introduction

Dimethyl fumarate (DMF), the methyl ester of fumaric acid, is a small molecule with immunomodulatory, anti-inflammatory and antioxidant effects [1]. Generally, fumaric acid esters (FAEs) have been used for several years in the treatment of psoriasis, a chronic inflammatory skin disease [2]. Then, FAEs have been discussed as therapeutic tools for autoimmune diseases beyond psoriasis [3], and their therapeutic efficacy has been proven also in the multiple sclerosis (MS) treatment [4]. DMF (Tecfidera^®^) was licensed as the first oral first-line therapy for relapsing-remitting multiple sclerosis (RRMS) [5,6] due to its immunomodulatory and neuroprotective effects and its favorable benefit–risk profile [4,7,8]. DMF administration leads to a reduction in both relapses and the progression of disability [9].

MS is a demyelinating autoimmune disease of the central nervous system (CNS) characterized by a strong inflammatory state and a selective destruction of myelin [10]. MS is the most common disabling neurological disease of young adults, with symptom onset generally occurring between the ages of 20 to 40 years [11] and affects approximately 2.8 million people worldwide [12]. About 85% of MS patients are diagnosed with RRMS disease course, which is characterized by the occurrence of relapses at irregular intervals with complete or incomplete neurological recovery.

The multifactorial working mechanisms by which DMF exerts its therapeutic effects in MS are not yet fully unraveled: mainly, DMF effects appear to be mediated by the activation of the pathway of the transcription factor nuclear factor erythroid 2 (NF-E2)-related factor 2 (Nrf2) [1]. Downstream of Nrf2, a cascade of antioxidant genes contributes to the anti-inflammatory process, reducing the levels of oxidative stress [13].

The drugs in use in the treatment of the MS, including DMF, are able to counteract the inflammation and, in the case of DMF, reduce the formation of new lesions [14,15]. However, the specific effects exerted by DMF on oligodendrocytes (OLs), which represent the most affected cell population in MS, have not yet been elucidated and need to be further investigated [16]. A recent study showed that DMF significantly alters the metabolism of OLs in a time-dependent manner, protecting them from oxidative stress [17].

OLs are the myelinating cells of the CNS, originating from finely regulated cellular specification processes. The most immature stage is represented by oligodendrocyte precursor cells (OPCs), which present a typical bipolar morphology. This cellular stage is followed by Pro-OLs/immature OLs, which are characterized by a branched morphology. This stage is preparatory to the achievement of the mature stage (mature OLs), characterized by a more pronounced branched morphology and by the production of myelin proteins, necessary to start the process of myelination of different axonal segments [18,19]. During their maturation, it is possible to identify different markers of the oligodendroglial lineage specific to the different stages of development. The OPCs express genes including *PDGFR*, *Vimentin* and *A2B5*. The Pro-OLs represent the first stage in which the *O4* gene is activated, then their expression will remain active until the mature OL stage. Immature OLs are identified by the expressions of *Gal4, O4* and *CNP*, while mature OLs, in addition to the already activated genes (O4 and *GalC*), will be characterized by the expression of genes for myelin proteins such as *MBP, MAG* and *MOG* [20].

OLs are highly vulnerable to oxidative stress produced during neuro-inflammation. There is now evidence that inflammation is actively involved in the pathogenesis of various neurodegenerative diseases, including MS [21,22,23]. Microglia are the innate immune component of the CNS. In healthy conditions, microglia support OPCs and mature OLs by secreting trophic factors promoting the myelination. After injury, microglia release pro-inflammatory cytokines, amplifying together with the astrocytes, the inflammatory process. In neurodegenerative diseases such as MS, a hyperactivation of microglia is observed; the increased levels of cytokines, as interleukin IL-6 and TNFα, are known to negatively influence OL survival and, consequently, the myelination process [24,25].

Experiments conducted in vitro showed that the release of cytotoxic effectors, following the stimulation of microglia with Lipopolysaccharide (LPS), damaged OPCs and OLs. OPCs differentiation was inhibited by oxidative agents and the subsequent release of TNFα [26,27,28]. The aim of the present work has been to evaluate the effects of DMF on in vitro Oli neu cells, oligodendrocyte lineage cells (OLCs) obtained from the stabilization of primary culture enriched in OLs and their precursor cells obtained from the brain of mouse embryos [29,30]. In particular, we have evaluated DMF anti-inflammatory properties and its possible action in promoting OLCs survival and differentiation. Several studies have shown that Oli neu cells represent a good model to study mechanisms associated with OL differentiation and myelination, even in neurodegenerative disorders [31]. Oli neu have been used as a model to characterize the expression of Connexins (Cx) involved in the CNS myelination process, such as Cx29, Cx47 and Cx32 [32], and as a model to study OL differentiation [33].

The results obtained demonstrate that DMF counteracts oxidative stress and lipid alterations directly on the OLCs, significantly reduces their proliferation and promotes their survival and differentiation.

## 2. Results

### 2.1. Characterization of Oli Neu Cells

**Figure 1:**
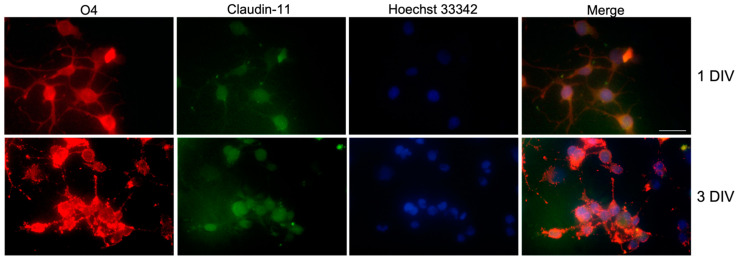
Immunocytochemistry showing the immunostaining for OL marker O4 (in red) and the myelin-associated protein Claudin-11 (in green) in Oli neu cells maintained for 1 and 3 DIV. Hoechst 33342 was used for nuclei staining. Scale bar: 20 μm.

The expression of OL markers in the Oli neu cell line was evaluated by immunocytochemistry. Cells were immunostained for O4, an antigen on the surface of OLs, which is commonly recognized as a specific marker of the pro-OLs [18], and for Claudin-11, a CNS-specific protein involved in the tight junctions of myelin sheaths in mice [34]. Oli neu cells expressed both O4 and Claudin-11 (Figure 1) at different time points of cultures (1–3 DIV; days in vitro), confirming the OL phenotype of the Oli neu cells and demonstrating that this cell model is appropriate for studying OLs in vitro.

### 2.2. Anti-Inflammatory Properties Mediated by DMF in Oli Neu Cells

**Figure 2:**
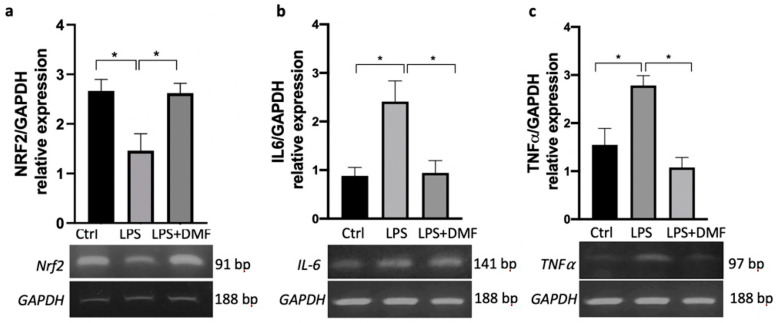
RT-PCR analysis of (**a**) *Nrf2*, (**b**) *IL-6* and (**c**) *TNFα* transcripts expressed in Oli neu cells treated with LPS 1 μg/mL and with LPS 1 μg/mL + DMF 25 μM for 24 h. The control consists of the vehicle in which DMF was dissolved (DMSO). GAPDH was used as housekeeping gene. The gel figures are representative of three independent experiments. Densitometric analysis was obtained from the average ± SEM of three independent experiments. One-way ANOVA test followed by the Tukey multiple comparison post-test was used to statistically compare the different experimental conditions (* *p* < 0.05).

FAEs are involved in the activation of the Nrf2 factor transcriptional cascade [35]. This pathway is implicated in cellular defense mechanisms by upregulating genes involved in antioxidant and cytoprotective responses [36]. In order to demonstrate these effects of DMF in our cell model, we evaluated Nrf2 gene expression in DMF-treated Oli neu cells after inducing an inflammatory environment upon LPS treatment (1 μg/mL) 2 h before the DMF (25 µM). LPS was chosen as the inflammatory molecule because several studies have shown that OLCs are able to respond to LPS, triggering an inflammatory response through binding to Toll-Like Receptor 4 (TLR4) [37,38]. After LPS stimulation, the levels of Nrf2 transcript are significantly reduced compared with control condition (Figure 2a), while DMF-treated cells showed an increase in Nrf2 transcript levels compared with cells treated with LPS alone (Figure 2a). This result confirms that DMF activates protective mechanisms from inflammatory events also in Oli neu cells. OLs are extremely sensitive to the inflammation and oxidative stress that may negatively impact OL survival [39]. Therefore, we evaluated the anti-inflammatory effects of DMF in Oli neu cells after stimulation with LPS, analysing the gene expression of the pro-inflammatory cytokines IL-6 and TNFα. RT-PCR analysis showed that both *IL-6* and *TNFα* transcript levels were upregulated after LPS treatment and that DMF significantly downregulated their expression compared with LPS treatment (Figure 2b,c).

### 2.3. Anti-Proliferative Effect of DMF on Oli Neu Cells

**Figure 3:**
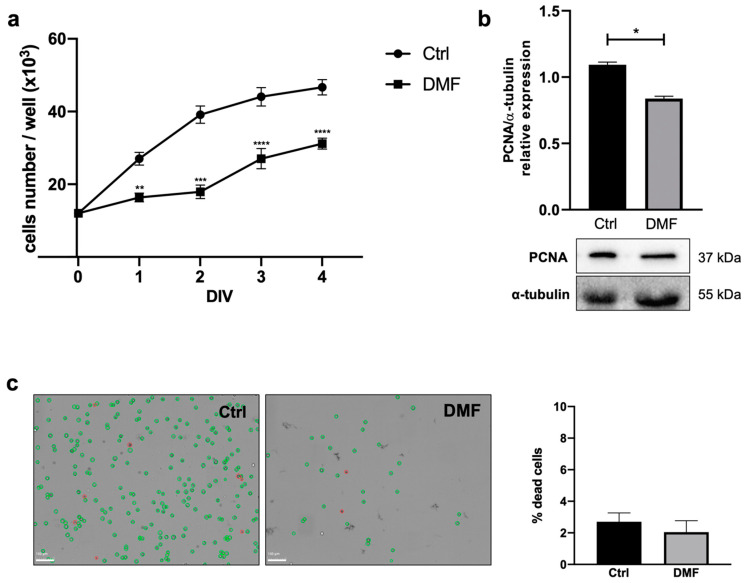
(**a**) Analysis of Oli neu cells growth by MTT assay in the control condition (DMSO) and after DMF 25 μM treatment (1, 2, 3, 4 DIV; days in vitro). Data are presented as mean ± SEM of three independent experiments performed in quadruplicate. Student’s *t*-test was used to statistically compare vehicle and treatment condition at each different experimental time (** *p* < 0.01, *** *p* < 0.001, **** *p* < 0.0001). (**b**) Representative western blot for PCNA protein expression in Oli neu cells in control (DMSO) and after 72 h of 25 μM DMF treatment. α-tubulin was used as internal reference protein. Densitometric analysis showed in the graph was obtained from three independent experiments. Student’s *t*-test was used to statistically compare the different experimental conditions (* *p* < 0.05). (**c**) Cell viability was assessed by cell counting. Oli neu cells were plated in triplicate, treated with vehicle (DMSO) or DMF 25 μM. After 72 h of treatment, cells were counted by LUNA-FX7™ using trypan blue dye, as shown by the representative images (live cells in green; dead cells in red). The graph shows the percentage of dead cells in the two experimental conditions; it is representative of three independent experiments. Student’s t-test was used to statistically compare the different experimental conditions (n. s. *p* = 0.4216).

The MTT assay is commonly used to evaluate cell growth [40,41,42]; for this purpose, it was used to examine the possible effects of DMF on Oli neu cell growth. We evaluated the effect of 25 μM DMF on Oli neu cells after 1-2-3-4 DIV of treatment. We found that DMF treatment significantly reduced cell growth, compared to the control condition (Figure 3a). This result was further supported by the reduction of Proliferating Cell Nuclear Antigen (PCNA) protein expression in Oli neu cells treated with DMF 25 μM for 72 h, as indicated by Western blot analysis (Figure 3b). In fact, PCNA is specifically expressed in actively replicating cells; it is a cofactor of DNA polymerase δ and increases the synthesis of the guide-strand during DNA replication [43]. To demonstrate that the arrest of cell growth after DMF treatment was not caused by increased cell death, we evaluated the cell viability after 72 h of DMF 25 μM treatment by trypan blue staining with LUNA-FX7™ Automated Cell Counter. As shown in the graph and in the representative cell count images (Figure 3c), albeit a reduced cell number after DMF treatment was evident, any change in the percentage of dead cells was observed compared with the control condition.

### 2.4. DMF Influences Oli Neu Cell Morphology and Differentiation

**Figure 4:**
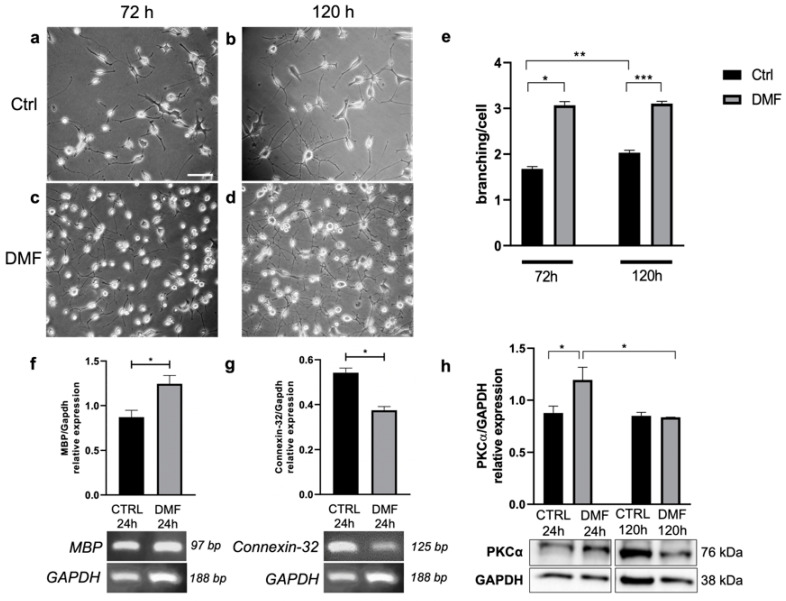
Representative images of Oli neu cells after 72 h or 120 h in the control condition (**a**,**b**) or after 25 μM DMF treatment (**c**,**d**)**.** Scale bar: 40 μm. (**e**) In the graph are reported the number of branchings/cells in different experimental conditions at 72 h and 120 h of treatment. Number of cells analysed at 72 h = 205 (in Ctrl DMSO condition) and 200 (in DMF condition); number of cells analysed at 120 h = 590 (in Ctrl DMSO condition) and 639 (in DMF condition). Student’s *t*-test was used to statistically compare Ctrl and DMF treated cells at 72 h of treatment (* *p* < 0.05; ** *p* < 0.01; *** *p* < 0.001). (**f**) RT-PCR analysis of *MBP* and (**g**) *Connexin-32* transcript levels in Oli neu cells in Ctrl (DMSO) or upon 24 h of 25 μM DMF treatment. *GAPDH* was used as housekeeping gene. The gel figures are representative of three independent experiments. Densitometric analysis was obtained from the average ± SEM of three independent experiments. Student’s *t*-test was used to statistically compare the different experimental conditions (* *p* < 0.05). (**h**) Western blot analysis of PKCα expression in Oli neu cells in Ctrl (DMSO) or upon 24 h or 120 h of 25 μM DMF. GAPDH was used as an internal reference protein. The figures are representative of three independent experiments. Densitometric analysis was obtained from three independent experiments. Student’s *t*-test was used to statistically compare the different experimental conditions (* *p* < 0.05).

To evaluate the effect of DMF treatment on Oli neu cell morphology, the reorganization of the cytoskeleton leading to the extension of cellular processes was investigated. After 72 h and 120 h of treatment with DMF 25 μM or vehicle (DMSO, ctrl), the morphology of Oli neu cells was analysed by counting the number of processes emitted by each cell in both experimental conditions (Figure 4a–d). Oli neu cells as well as the OPCs undergo a progressive and spontaneous differentiation if cultured in the presence of thyroid hormones [44]. In line with this characteristic, we observed a progressive increase in the number of processes in the control condition, comparing Oli neu cells at 72 and 120 h of culture. However, in the presence of DMF, it is possible to observe a significant increase in Oli neu cell processes branching both after 72 h and 120 h of treatment, compared to the control conditions (Figure 4e). The above-mentioned findings regarding the anti-proliferative action of DMF and the increase in the average number of processes per cell after long-time treatment with DMF led us to investigate the possible role of DMF in stimulating Oli neu cell differentiation. Therefore, we analysed the transcriptional expression of two major myelin components, Myelin Basic Protein (MBP) and Connexin-32 (CX-32). MBP is an extrinsic protein of the myelin sheath; it is expressed in mature and myelinating OLs [18]. By RT-PCR analysis, we found that 24 h treatment with DMF 25 μM induced an increase of *MBP* expression compared with the control (DMSO) (Figure 4f). On the other hand, CX-32 is a myelin protein typically localized in non-compact myelin regions [41]. By RT-PCR analysis, we observed that the treatment with 25 μM DMF for 24 h induced a significant reduction in the *Cx-32* expression compared with the control (Figure 4g). A recent study supported the involvement of protein kinase Cα (PKCα) in glial cell maturation and myelination [45]. The activation of PKCα has been found to drive a gliogenesis program associated with the modulation of several biological processes, including remodeling of the cytoskeleton, which is critical in the formation of differentiation processes [45,46,47]. Western blot analysis showed that 24 h of treatment with 25 μM DMF induced a significant increase in PKCα expression compared with the control (DMSO) (Figure 4h), supporting the hypothesis of a pro-differentiative effect of DMF in Oli neu cells. No differences were observed after 120 h of treatment (Figure 4h).

### 2.5. Antioxidant Effects of DMF on Oli Neu Cells

**Figure 5:**
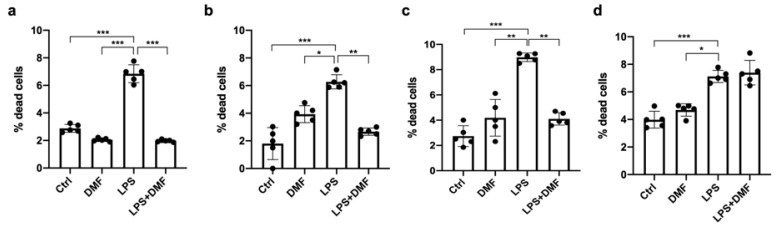
Cell viability in Oli neu cells after LPS and DMF treatment. Oli neu cells were plated in sextuplicate and treated with vehicle (DMSO) (Ctrl), DMF 25 μM, LPS 1 μg/mL and LPS+DMF. After (**a**) 24 h, (**b**) 48 h, (**c**) 72 h and (**d**) 120 h of treatment, the cells were counted by LUNA-FX7™ using trypan blue dye. The graph, showing the percentage of dead cells, was obtained from the average ± SEM of five independent experiments. One-way ANOVA test followed by the Tukey multiple comparison post-test was used to statistically compare the different experimental conditions (* *p* < 0.05; ** *p* < 0.01; *** *p* < 0.001).

The antioxidant properties of DMF are widely known [48], as well as the specific increase of reactive oxygen species (ROS) intracellular levels induced by LPS [49,50]. To more accurately interpretate the data regarding ROS accumulation, we first evaluate the effect of LPS on the Oli neu cell viability in the presence or absence of DMF. The cells were treated with 25 μM DMF, LPS 1 μg/mL and LPS + DMF for 24, 48, 72 and 120 h. As observed in the Figure 5, the percentage of dead cells treated with LPS was significantly higher than untreated cells (Ctrl) and DMF-treated cells. As shown in the graphs in the Figure 5a–c, until 72 h DMF was able to counteract the cytotoxic effect of LPS on the Oli neu cells. This effect was not observed at 120 h (Figure 5d).

**Figure 6:**
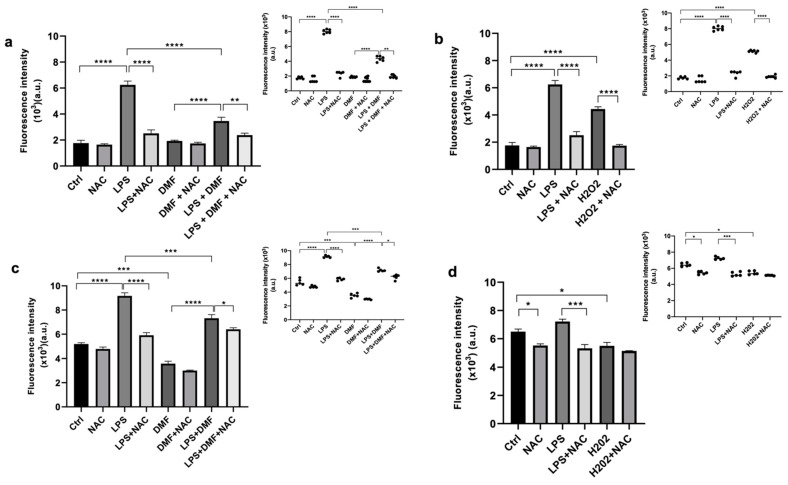
Measurement of ROS levels by DCFDA staining in Oli neu cells under the following experimental conditions: LPS 1μg/mL, DMF 25 μM and LPS 1 μg/μL + DMF 25 μM, in presence or absence of NAC (10 μM). Cells were used after 1 DIV (**a**,**b**) or 5 DIV (**c**,**d**) from seeding to evaluate the responses of OPC or pro-OLs cells at different experimental conditions. H_2_O_2_ (20 μM) treatment was used as a positive control (**b**,**d**). The data are the average ± SEM of five independent experiments conducted in sextuplicate. One-way ANOVA test followed by the Turkey multiple comparison post-test was used to statistically compare the different experimental conditions (* *p* < 0.05; ** *p* < 0.01; *** *p* < 0.001; **** *p* < 0.0001).

Then we analysed the effect of DMF treatment on LPS-stimulated Oli neu cells after 1 DIV (Figure 6a,b) and 5 DIV (Figure 6c,d) in culture. The intracellular ROS production was evaluated by dichloro-dihydro-fluorescein diacetate (DCFDA) staining. The use of the ROS scavenger N-acetyl-l-cysteine (NAC) allows for counteracting the ROS increase; this molecule was used as a control of ROS induction. As expected, LPS treatment strongly increased ROS levels in Oli neu cells compared with the control. Instead, in the presence both of NAC and DMF, the LPS-induced increase of ROS levels was significantly reduced (Figure 6a,c). On the other hand, as a positive control of the experiment, the Oli neu cells were also treated with H_2_O_2_ (20 μM), which is known to trigger oxidative stress [51]. As shown in figure Figure 6b,d, H_2_O_2_ caused a significant increase of ROS levels, albeit the increase was lower than in LPS treated cells. NAC was able to counteract the H_2_O_2_-induced ROS increase.

### 2.6. DMF Effects on Lipid Content of Oli Neu Cells

**Figure 7:**
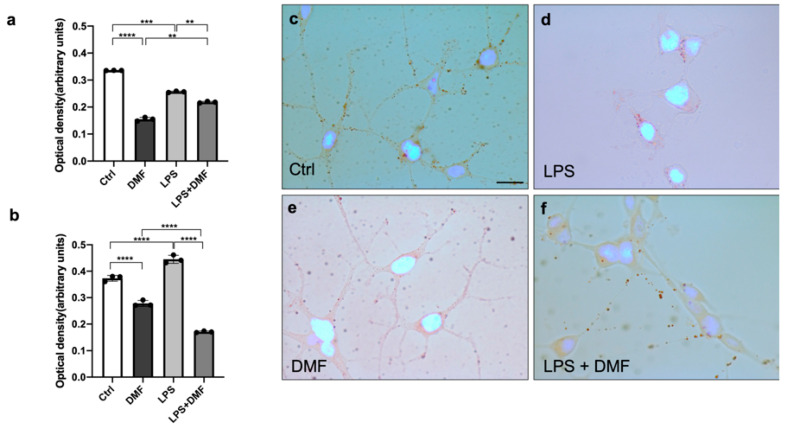
Lipid accumulation assay using Oil Red O staining. The graphs show the optical density relative to the number of LDs measured in the Oli neu cells in the following experimental conditions: untreated cells (Ctrl), LPS 1 μg/mL, DMF 25 μM and LPS 1 μg/μL + DMF 25 μM, after 48 h (**a**) and 120 h (**b**) of treatment. The data were obtained as the average ± SEM of three independent experiments performed in sextuplicate. One-way ANOVA test followed by the Turkey multiple comparison post-test was used to statistically compare the different experimental conditions (** *p* < 0.01; *** *p* < 0.001; **** *p* < 0.0001). Representative images of Oli neu cells maintained for 48h in culture: vehicle-treated cells (Ctrl) (**c**), LPS 1 μg/mL (**d**), DMF 25 μM (**e**) and LPS 1μg/mL + DMF 25 μM (**f**) treatments. Scale bar: 10 μm.

Many neurodegenerative diseases are characterized by high levels of oxidative stress [52], and it is well known that high levels of ROS determine an increase in lipid synthesis and lipid droplets (LDs) formation in glial cells [53]. Following the experiments carried out to detect ROS after inflammatory stimulation, the effect of DMF treatment on LDs production in Oli neu cells was also evaluated (Figure 7). Cells were treated with LPS 1 μg/mL and LPS + DMF 25 μM for 48 h and 120 h to compare the number of LDs in Oli neu cells at different stages of OL differentiation [OPCs (48 h) and pro-OLs (120 h)]. By Oil Red O staining, quantitative analysis was performed to measure LD levels in Oli neu cells. As shown in the Figure 7b, at 120 h after LPS stimulation, we observed an increase in LDs compared to the control condition. At both experimental times (Figure 7a,b), cells treated with DMF alone or with LPS + DMF showed a significant decrease in Oil Red O staining compared to untreated cells and LPS-treated cells, indicating a decrease in LD content. The reduction of LDs was also observed by a light field microscopy analysis in cells treated for 48 h (Figure 7c–f). A decrease in the number and size of LDs was observed in DMF-treated cells compared with controls. The same result was observed at 120 h (data not shown).

Since the core of LDs consists of predominantly triglycerides (TG) and cholesterol ester (Chol-E) [54], we proceeded to observe a possible decrease of these two lipid species using thin-layer chromatography (TLC).

**Figure 8:**
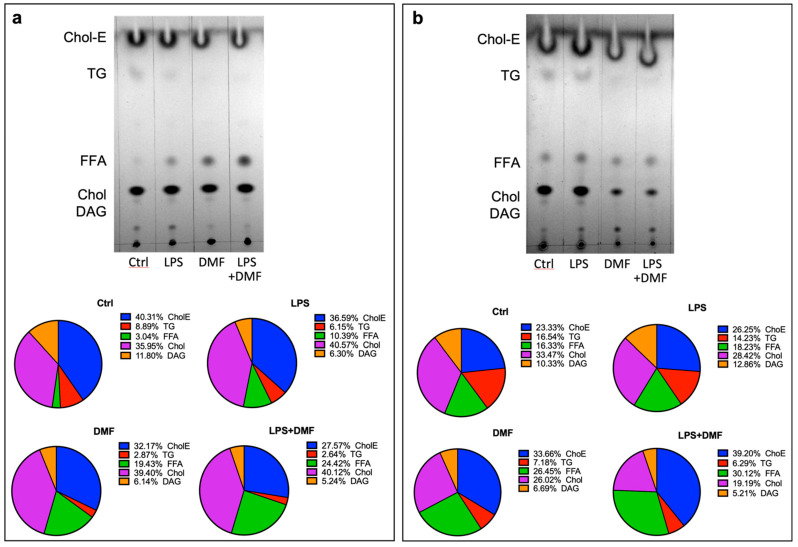
LDs contents were evaluated by TLC in Oli neu cells at different experimental conditions: cells treated with vehicle (Ctrl), LPS 1 μg/mL, DMF 25 μM and LPS 1 μg/μL + DMF 25 μM after 48 h (**a**) and 120 h (**b**). Representative images were selected from three independent experiments. Pie charts represent the percentages of the different lipid species analysed (Chol-E, TAG, FFA, Chol, DAG) under the four experimental conditions.

Indeed, through TLC experiments, it was possible to analyse the content of different neutral lipid species, such as Chol-E, TG, free fatty acids (FFA), Chol and diacylglycerol (DAG) in Oli neu cells (Figure 8). Cells were treated with LPS 1 μg/mL, DMF 25 μM or LPS + DMF for 48 h (Figure 8a) and 120 h (Figure 8b). TLC showed no significant changes in the lipid species distribution between control (DMSO)- and LPS-treated cells. Conversely, relevant changes were observed in DMF-treated cells at both experimental times. A different distribution of lipid species was observed in the DMF and LPS + DMF conditions compared to the control and the LPS conditions. In the presence of DMF, it was evident an increase in FFA and a decrease in TG. Moreover, after 120 h of DMF treatment, a decrease of Chol-E was also detected.

The decrease in these two lipid species (TG and Chol-E) caused by DMF treatment is coherent with the decrease in LDs observed with Oil Red O staining.

## 3. Discussion

The common anti-inflammatory drugs used for the MS treatment cause a reduction of the neuro-inflammation, favoring both neurons and OLs survival, but it is not known whether it may support the re-myelination. However, DMF shows beneficial effects on myelin in patients with MS, but it has not yet been elucidated whether this is due to a direct effect on OLs or it is consequential to the action of DMF on inflammatory microenvironment [17].

In the present study, the Oli neu cell line was chosen as the cell model for our in vitro studies. It presents OL marker O4 and the OL-specific myelin protein Claudin-11, as demonstrated by immunocytochemistry studies (Figure 1). As the OLs, Oli neu cells also undergo a progressive and spontaneous differentiation, if maintained in the presence of thyroid hormones [44]. For this reason, some of our investigations were performed at two different experimental time points, the 24 and 120 h of cell culture, in order to compare the effects of DMF on Oli neu cells at an early and a pro-differentiated OLs stages, respectively.

Previous studies have shown that OPCs are able to respond to LPS, triggering an inflammatory response through binding to TLR4 and activating the transcription of pro-inflammatory cytokines such as IL-6 and TNFα [37,38]. Given the close relationship between inflammatory response and ROS production, LPS was used to stimulate the in vitro inflammatory microenvironment useful to study the effects of DMF on experimental inflammation.

The ability of DMF to positively modulate the *Nrf2* expression also in Oli neu cells was confirmed by RT-PCR analysis, demonstrating that DMF stimulates an increase in *Nrf2* transcript expression respective to LPS that, as expected, has an inhibitory effect on its expression (Figure 2a).

DMF was also able to counteract the production of IL-6 and TNFα, after LPS stimulation, directly in Oli neu cells (Figure 2b,c). This confirms the well-known anti-inflammatory properties attributed to DMF, and it also extends its effects to OLCs.

OLs are highly vulnerable to oxidative stress. Cytokines play a central role in inflammation, demyelination and neurodegenerative processes. During inflammatory demyelinating disease, they produce reactive species of oxygen able to affect OLs viability [21].

In this study we demonstrated that DMF induced a significant reduction of Oli neu cell growth, studying their proliferation rate by MTT assay at different experimental times ranging from 1 to 4 DIV (Figure 3a). We ensured that the proliferation rate reduction did not correlate with an increase in cell death (Figure 3c). Since we did not observe any change in the percentage of dead cells between vehicle—and DMF—treated cells, we concluded that DMF indeed induced a slowdown of OL proliferation without affecting cell viability. This result was also confirmed by the decreased expression of PCNA in DMF-treated cells (Figure 3b); PCNA in fact is mainly expressed by proliferating cells, playing an essential role in DNA replication and repair. These data led us to speculate that DMF may promote the differentiation process at the expense of cell proliferation. Therefore, we carried out experiments aimed at evaluating the differentiation process of Oli neu cells, treated or not with DMF. DMF treatment was shown to determine an increase in the average of number of processes emitted per cell (Figure 4a–e). This finding supported the hypothesis that DMF may support the Oli neu differentiation. However, we observed a general trend towards a time-dependent increase in processes emitted per cell, even in the control condition. We assumed that Oli neu cells can spontaneously differentiate in vitro as well as OPCs when they were cultured in presence of thyroid hormones [41]; however, it was clear that DMF treatment may accelerate this process.

OLs maturation process involves the acquisition of myelinating properties; therefore, we evaluated the expression levels of myelin proteins such as MBP and CX-32 (Figure 4f,g, respectively). MBP is an extrinsic myelin sheath protein and is typically expressed by mature oligodendrocytes, whereas CX-32 is a gap junction-forming membrane protein and is generally expressed in non-compact myelin domains [41]. We found that DMF treatment induced an increase in *MBP* transcript expression, while it reduced the transcript levels of *CX-32*. Another factor whose role in glial cell maturation and myelination has recently been discovered is PKCα [45]. Western blot analysis showed that after 24 h of treatment with DMF, an increase in PKCα protein expression was observed in Oli neu cells compared to the control condition (Figure 4h). This trend was not observed after 120 h of DMF treatment. This could suggest that DMF accelerate OL maturation through PKCα upregulation in particular on OPCs.

In order to test the antioxidant properties of DMF, the levels of intracellular ROS levels were assessed by staining with DCFDA (Figure 6). According to the literature, LPS, stimulating the inflammatory environment [43], increased the level of ROS in Oli neu cells and NAC, a scavenger of ROS, was able to counteract the LPS-induced increase of ROS levels. The analysis showed that DMF treatment reduced the levels of oxidative stress counteracting the LPS effects directly on Oli neu cells. These data confirmed the already-known antioxidant properties of the molecule. DMF maintained the same properties both when the cells were in an early stage (24 h after seeding) and in a later stage (120 h after seeding) of culture, when the cells appear more differentiated.

In recent years, great progress has been made in the study of lipid homeostasis in the CNS. In particular, several studies are making a major contribution to the understanding of the role of LDs in various diseases, including neurodegenerative diseases. LDs may affect cellular physiology and function in the CNS [44]. Inflammation is one of the causes of the accumulation of LDs. LPS has been shown to increase the number and size of LDs in microglia [45]. Therefore, high levels of ROS increase lipid synthesis and LDs formation. LDs are associated with protection from ROS and sequestration of toxic lipophilic species [46,47]. After ROS analysis, we analysed the accumulation of LDs by Oil Red O staining in the control condition and after providing LPS, DMF or both (Figure 7). As expected, quantitative and microscopic analysis showed an accumulation of LDs after LPS treatment. This accumulation is significantly reduced by DMF treatment, indicating that the number of the LDs follows the trend of ROS production. Thus, DMF in Oli neu cells was able to reduce both the levels of oxidative stress and the number of LDs produced, under both basal conditions and after the inflammatory stimulus. The cell viability experiment (Figure 5) allowed us to demonstrate that the reduction of ROS and LDs in Oli neu cells treated with LPS + DMF compared to cells treated only with LPS is an effective reduction, and it was not dependent on cell death. In fact, until 72 h of treatment, DMF is able to reverse the increase in mortality of Oli neu cells –LPS induced. Moreover, the antioxidant and anti-inflammatory effects of DMF were found to be more significant after 120 h of treatment, suggesting that the more differentiated cells present more lipids, accordingly with the increased branching of the OLC processes and the production of more membranes necessary during the myelination process. TLC analysis confirmed the reduction of LDs amount (Figure 8). Indeed, after 48 h of DMF treatment, a reduction in TG and Chol-E, the two major components of LDs, was observed in both basal and inflammatory conditions. By TLC analysis, it was also interesting to observe that DMF treatment at 48 and 120 h resulted in a greater number of FFA compared to the control, under both basal and inflammatory conditions. In general, OLs require rapid access to large amounts of lipids to myelinate the axons [48,49]. A recent study has shown that OLs depend on the endogenous de novo synthesis of fatty acids to support OL remyelination [50].

## 4. Materials and Methods

### 4.1. Cell Culture

The Oli neu cell line was obtained from Dr Trotter’s laboratory. This is a primary culture enriched in OLs and their precursor cells, derived from the brains of 15-day-old mouse embryos. Neurons were removed by immunocytolysis mediated by the complement. The line was then immortalized by transfection with a retroviral vector, which encodes for a hybrid protein between the outer domain of the human EGF receptor and neu tyrosine kinase [29,30]. Cells were cultured on poly-l-Lysine-coated dishes (100 μg/mL; Sigma-Aldrich, St. Louis MO, USA), in Dulbecco’s Modified Eagle’s Medium (DMEM; Sigma-Aldrich, St. Louis, MO, USA) supplemented with 50 μg/mL of streptomycin, 50 IU/mL of penicillin, 2 mM of glutamine (Immunological sciences, Rome, Italy), 25 μg/mL gentamicin (Sigma-Aldrich, St. Louis, MO, USA), 2% horse serum (Sigma-Aldrich, St. Louis, MO, USA), 10 μg/mL transferrin (Sigma-Aldrich, St. Louis, MO, USA), 10 μg/mL insulin (Sigma-Aldrich, St. Louis, MO, USA), 100 μM putrescine (Sigma-Aldrich, St. Louis, MO, USA), 200 nM progesterone (Sigma-Aldrich, St. Louis, MO, USA), 500 nM triiodothyronine (Sigma-Aldrich, St. Louis, MO, USA), 220 nM sodium selenite (Sigma-Aldrich, St. Louis, MO, USA) and 520 nM L-thyroxine (Sigma-Aldrich, St. Louis, MO, USA). The cell line was maintained at 37 °C in a humidified incubator in an atmosphere with 10% CO_2_.

### 4.2. Pharmacological Treatment

Dimethyl fumarate (DMF, Sigma-Aldrich, St. Louis, MO, USA) is an oral therapeutic agent, commonly used in RR-MS patients. It has been proved to have immunomodulatory, anti-inflammatory and antioxidant effects [48]. DMF was dissolved in DMSO (Sigma-Aldrich, St. Louis, MO, USA). Twenty-four hours after seeding, cells were treated with 25 μM DMF (Sigma-Aldrich, St. Louis, MO, USA), whereas controls are cells treated with an equal volume of DMSO, used to dissolve the DMF. H_2_O_2_ used as a positive control of ROS induction was used at a final concentration of 20 μM.

### 4.3. Cell Viability Assay

Cell proliferation was assessed by colorimetric assay based on 3-(4.5-dimethyl thiazol 2-y1)-2.5-diphenyl tetrazolium bromide (MTT, Sigma-Aldrich, St. Louis, MO, USA) metabolization, according to the protocol optimized by Mosmann [55]. Cells were seeded on poly-l-Lysine-coated 96-well plates at a density of 1.2 × 10^4^ cells/well. After 24 h, cells were treated with DMF 25 μM (or DMSO only) at different experimental time points (ranging from 24 to 96 h). MTT was dissolved in PBS at 5 mg/mL to obtain the 10X stock solution. The stock solution was diluted (1X), added in each well and then incubated at 37 °C for 3 h. A solution of isopropanol, HCl 0.04 M and 1% Triton X-100 was added to all wells and mixed thoroughly to mechanically dissolve the dark blue crystals. For each well, the optical density (OD) at 570 nm was measured by Multiskan FC (Thermofisher Scientific, Waltham, MA, USA).

The possible toxicity of DMF and LPS on Oli neu cells was assessed by a viability assay using trypan blue staining. OLCs were treated with DMF (and vehicle, DMSO), LPS 1 μg/μL + DMSO, DMF + LPS for 24, 72 and 120 h. The floating and adherent cells were collected and stained with trypan blue, a dye that stains only dead cells. The percentage of dead cells was analysed by LUNA-FX7™ Automated Cell Counter (Logos Biosystems, Gyeonggi-do, South Korea).

### 4.4. Morphological Analysis

Cells were seeded on poly-l-Lysine-coated 60 mm-dishes at a density of 3.5 × 10^5^ cells. After 24 h from seeding, cells were treated with DMF 25 μM (or DMSO only). After 72 h and 120 h treatments, 3 bright-field images for each experimental condition were analysed by counting the processes emitted by each cell with ImageJ software imaging software (NIH, Bethesda, MD, USA). The experiments were performed in triplicate.

### 4.5. Total RNA Extraction and RT-PCR Analysis

Total RNA was extracted using TRI Reagent^®^ (Sigma-Aldrich, St. Louis, MO, USA), according to the manufacturer’s instructions. RNA was quantified at NanoDrop™ 2000 spectrophotometer (Thermo Fisher Scientific, Waltham, MA, USA). For each sample, 1 µg of total RNA was reverse transcribed using 5X All-In-One RT MasterMix with AccuRT Genomic DNA Removal Kit (Applied Biological Materials Inc., Richmond, CA, USA), according to the manufacturer’s protocol. For each sample, primers and GoTaq^®^ Green Master Mix (Promega Italia, Milan, Italy) were added to 100 ng of cDNA. The expression of the transcripts was evaluated by semi-quantitative RT-PCR analysis, using the following primers:

*Nrf2:* forward 5′-GCCCTCAGCATGATGGACTT-3′

reverse 5′-TGTCTTGCCTCCAAAGGATGT-3′

*IL-6:* forward 5′-CCCCAATTTCCAATGCTCTCC-3′

reverse 5′-CGCACTAGGTTTGCCGAGTA-3′

*TNFα:* forward 5′-ATGGCCTCCCTCTCATCAGT-3′

reverse 5′-TTTGCTACGACGTGGGCTAC-3′

*MBP*: forward 5′-CCCCAGAGCTGGTGCTTTTA-3′

reverse 5′-GAGAACTCCTGCAGTCCCAC-3′

*Connexin-32:* forward 5′-TGGAAGAGGTAAAGAGACACAAGG-3′

reverse 5′-CGGGGTAGAGCAGCTAGAAGACA-3′

*GAPDH*: forward 5′-TGGCATTGTGGAAGGGCTCATGA-3′

reverse 5′-ATGCCAGTGAGCTTCCCGTTCAG-3′

### 4.6. Protein Extraction and Western Blot Analysis

Cells were homogenized in lysis buffer (Tris-EDTA 10 mM, 0.5% NP40 and NaCl 150 mM) containing a protease inhibitor cocktail (Sigma-Aldrich, St. Louis, MO, USA). After protein extraction, the total amount of protein was determined by a Pierce BCA Protein Assay Kit (Thermo Fisher Scientific, Waltham, MA, USA) according to the manufacturer’s protocol. The protein extracts were run on SDS-polyacrilamide gel (SDS-PAGE) and transferred to polyvinylidene difluoride (PVDF) sheets (Merck Millipore, Darmstadt, Germany). Membranes were blocked in 5% non-fat milk powder (Sigma-Aldrich, St. Louis, MO, USA) in PBS containing 0.1% Tween-20 and then incubated overnight at 4 °C with one of the following primary antibodies: anti-PCNA (1:700, Sigma-Aldrich, St. Louis, MO, USA), anti-PKCα (dilution 1:2000, Immunological Science, Milan, Italy, AB-84289), anti-α tubulin (1:500, Immunological Science, Milan, Italy) and anti-GAPDH (1:300, Immunological Science, Milan, Italy). α-tubulin and GAPDH were used as reference proteins for loading control. The blots were washed three times with PBS + 0.1% Tween-20, then incubated with secondary antibody for 1 h at room temperature (RT): anti-rabbit horseradish peroxidase (1:10,000, Promega, Madison WI, USA) or anti-mouse horseradish peroxidase (1:10,000, Immunological Science, Milan, Italy). Immunoreaction was revealed by ECL chemiluminescence reagent (Immunological Science, Milan, Italy). The bands were detected by exposition to Chemidoc (Molecular Imager ChemiDoc XRS + System with Image Lab Software; Bio-Rad, CA, USA).

### 4.7. Immunocytochemistry

Oli neu cells were plated on poly-l-Lysine-coated 12 mm-coverslips at a density of 3 × 10^4^ cells/well. After 24 h or 120 h, cells were fixed with 4% paraformaldehyde for 20 min at RT and washed three times in PBS. The cells were permeabilized by incubation in PBS containing 0.1% Triton X-100, 10% NGS (Thermo Fischer, Walthman, MA, USA), 1% BSA (Sigma-Aldrich, St. Louis, MO, USA) for 45 min at RT. The cells were then incubated overnight at 4 °C with anti-O4 (10 µg/mL, R&D Systems, Inc. NE Minneapolis, MN, USA) and anti-Claudin-11 (1:100, Immunological Science, Milan, Italy) antibodies, diluted in PBS with 0.1% Triton X-100, 1% NGS and 1% BSA. The cells were then washed in PBS and incubated for 1 h and 30 min at RT with goat anti-mouse Alexa 594-conjugated and goat anti-rabbit Alexa 488-conjugated (Immunological Science) secondary antibodies, diluted 1:100 in PBS with 0.1%, 1% NGS and 1% BSA. Finally, cells were incubated with Hoechst 33432 (1:1000 in PBS, Thermo Scientific, Waltham, MA, USA) for 10 min at RT, for the nuclei counterstaining. At the end, coverslips were fixed on microscope slides with a PBS-glycerol (3:1; *v*/*v*) solution. The images were acquired with a Zeiss fluorescence microscope through the Zen lite software (Zeiss, Oberkochen, Germany).

### 4.8. ROS Detection Assay

ROS production was measured using dichloro-dihydro-fluorescein diacetate (DCFDA; Enzo Life Sciences, New York, NY, USA) used as an oxidation substrate.

Cells were seeded on poly-l-Lysine-coated 96-well plates at a density of 1 × 10^4^ cells/well. The next day, the cells were incubated with DMSO (as Ctrl), DMF 25 μM, H_2_O_2_ 20 μM, LPS 1 μg/μL + DMSO, DMF + LPS and H_2_O_2_ + LPS for 2 h.

The ROS scavenger N-acetyl-l-cysteine (NAC; Enzo Life Sciences, New York, NY, USA) at a final concentration of 10 μM was used to inhibit ROS production; it was added 45 min before the pharmacological treatments. Then, the medium was removed, and cells were incubated with 10μM DCFDA for 30 min. The same protocol was used in differentiating cells, 120 h after seeding. The fluorescence was read at wavelengths of 485 nm of excitation and 530 nm of emission using the Glomax Multi Detection System (Promega Italia, Milan, Italy).

### 4.9. Oil Red O Staining

The fat-soluble dye Oil Red O was used to detect lipid droplets (LDs). It has the appearance of a red powder soluble in isopropanol, which dissolves lipid molecules, turning them orange-red in color. Cells were seeded on poly-l-Lysine-coated 24-well plates at a density of 3 × 10^4^ cells/well. After 48 h or 120 h, the cells were fixed with 4% paraformaldehyde for 20 min at RT and washed five times in PBS in order to remove the dead cells. Then, cells were incubated with the working solution (Oil Red O: dH_2_O, *v/v*, 3:2) for 30 min in the dark and washed three times in PBS. Finally, cells were incubated with Hoechst 33342 (1:1000 in PBS, Thermo Scientific, Waltham, MA, USA) for 10 min at RT, for the nuclei counterstaining. At the end, coverslips were fixed on microscope slides with a PBS-glycerol (3:1; *v*/*v*) solution. The images were acquired with a Zeiss microscope through the Zen lite software (Zeiss, Oberkochen, Germany). A quantitative analysis can also be obtained from Oil Red O staining by measuring absorbance. Cells were seeded on poly-l-Lysine-coated 96-well plates at a density of 1 × 10^4^ cells/well. After 48 h or 120 h, cells were incubated with the working solution (Oil Red O: dH_2_O, *v/v*, 3:2) for 30 min in the dark. After 5 washes in dH_2_0 to remove excess of the dye, 50 μL of isopropanol has been added in each well and left stirring for 10 min at room temperature. LDs quantification is obtained by Clariostar Plus Microplate Reader (BMG Labtech, Ortenberg, Germania); the optical density (OD) was measured at 570 nm.

### 4.10. Analysis of Neutral Lipid Content by Thin-Layer Chromatography (TLC)

The content of neutral lipids under different treatment conditions was evaluated by TLC. Total lipids were extracted by the Bligh and Dyer procedure [56]. Lipids normalized for cell number in each experimental condition were loaded on a silica gel plate for separation by TLC. Neutral lipids were revealed with hexane/ethyl ether/acetic acid (70/30/1; *v/v/v*). After revelation, plates were uniformly sprayed with 10% cupric sulfate in 8% aqueous phosphoric acid and allowed to dry for 10 min at RT. Then the plates were placed at 145 °C for 10 min. Through TLC, it is possible to separate the different neutral lipid species, such as cholesterol ester (Chol-E), triglycerides (TG), free fatty acids (FFA), cholesterol (Chol) and diacylgrycerol (DAG).

Spots were revealed by exposition to Chemidoc (Molecular Imager ChemiDoc XRS + System with Image Lab Software, Bio-Rad, CA, USA), and their intensities were measured by densitometric analysis (ImageJ software, National Institutes of Health, Bethesda, MD, USA).

### 4.11. Statistical Analysis

Data were presented as the mean ± standard error of the mean (SEM). Student’s *t*-test or one-way ANOVA tests were used to evaluate statistical significance within the different samples. Results were considered statistically significant at *p* < 0.05 (*), *p* < 0.01 (**), *p* < 0.001 (***) and p < 0.0001 (****). Data analyses were performed with GraphPad Prism 8 (GraphPad Software, La Jolla, CA, USA).

## 5. Conclusions

An ideal molecule for the treatment of the demyelinating disease such as MS, for which currently there is no definitive cure, should be able to modulate immune system activity, counteract neuro-inflammation, protect OPCs and promote the re-myelination after damage. Our results confirmed the known anti-inflammatory and antioxidant properties of DMF, extending these effects also to Oli neu cells.

Interestingly we also demonstrate that DMF does not appear to be toxic for Oli neu cells, and it is able to induce a slowdown of cell proliferation in favor of differentiation in pro-myelinating OLs. Albeit these data required further investigation also in human OLs, we at least can conclude that DMF appears to combine both antioxidant effects and myelin pro-regenerative property.

## Data Availability

Not applicable.
